# Cleanliness Grades as Clinical Indicators of Vaginal Infection Burden in Women from Northern Madagascar: A Cross-Sectional Study

**DOI:** 10.3390/jcm15052008

**Published:** 2026-03-05

**Authors:** Daniel Kasprowicz, Franco Rajaomalala, Krzysztof Korzeniewski, Wanesa Wilczyńska

**Affiliations:** 1 Clinique Médicale Beyzym, Manerinerina RN6, Ambatoboeny District, Manerinerina 403, Madagascar; daniel.kasprowicz@icloud.com (D.K.);; 2 Department of Epidemiology and Tropical Medicine, Military Institute of Medicine—National Research Institute, 04-141 Warsaw, Poland; kkorzeniewski@wim.mil.pl

**Keywords:** bacterial vaginosis, Madagascar, *Neisseria gonorrhoeae*, *Trichomonas vaginalis*, vaginal biocenosis

## Abstract

**Background**: Bacterial vaginosis and vaginal dysbiosis represent major causes of morbidity among women in sub-Saharan Africa, yet data from Madagascar remain scarce. This study aimed to assess the prevalence and determinants of vaginal bacterial infections among women in northern Madagascar and to explore how vaginal microflora composition reflects broader aspects of reproductive health. **Methods:** A cross-sectional study was conducted in April 2024 among 159 women (15–80 years) attending a rural second-referral clinic in Manerinerina, Ambatoboeny District. Sociodemographic and hygiene data were obtained through structured questionnaires. Vaginal pH was measured in situ, and Gram-stained smears were evaluated using the Nugent scoring system. The presence of *Trichomonas vaginalis*, *Neisseria gonorrhoeae*, and *Candida* spp. was assessed microscopically. Associations were analyzed using Chi-square or Fisher’s exact tests, with *p* < 0.05 considered significant. **Results:** Abnormal vaginal flora was observed in 68.6% of women, including 43.4% with BV (Nugent 7–10) and 25.2% with intermediate flora. Elevated vaginal pH correlated strongly with higher Nugent scores (*p* < 0.01). *T. vaginalis* and *N. gonorrhoeae* were detected in 10.7% and 9.4% of women, respectively, and both were significantly associated with dysbiosis (*p* = 0.02 and *p* = 0.04). Poor hygiene practices, vaginal douching (79.1% vs. 64.5%; *p* = 0.04), and unsafe water sources (*p* = 0.04) were major behavioral and environmental determinants. **Conclusions:** Vaginal dysbiosis is highly prevalent among women in northern Madagascar and closely linked to modifiable hygiene behaviors and environmental conditions. In resource-limited settings, Gram-stained microscopy and Nugent scoring remain cost-effective tools for surveillance and patient care. Culturally adapted education, improved water access, and integration of low-cost diagnostics are essential for reducing the burden of vaginal infections in rural Madagascar.

## 1. Introduction

Bacterial vaginosis (BV) is a polymicrobial dysbiosis of the vaginal ecosystem characterized by a reduction or loss of *Lactobacillus* species and an overgrowth of anaerobic and facultative bacteria such as *Gardnerella vaginalis*, *Prevotella* spp., and *Mobiluncus* spp. [1,2]. The pathophysiology involves an increase in vaginal pH, decreased lactic acid production, and the disruption of protective hydrogen peroxide-producing *Lactobacillus* strains, which facilitates colonization by pathogenic bacteria [1,3]. Known risk factors include multiple sexual partners, vaginal douching, intrauterine device use, and cigarette smoking [4,5]. Globally, BV remains the most prevalent cause of vaginal discharge among women of reproductive age, with estimated prevalence ranging from 20 to 30% in the general population to as high as 50–60% in high-risk groups [1,2].

In sub-Saharan Africa, BV remains a significant burden to women’s reproductive health, contributing to adverse pregnancy outcomes and increased susceptibility to sexually transmitted infections. Population-based studies report BV in approximately 25–30% of women in the general population, while rates among high-risk groups such as female sex workers and women living with HIV may exceed 50–60% [6,7,8]. A pooled analysis of more than 37,000 women from 18 studies across the region confirmed BV as one of the most prevalent vaginal infections, with considerable heterogeneity between countries and subpopulations [7]. Socio-behavioral and environmental determinants—including poor hygiene, limited access to reproductive healthcare, high HIV prevalence, and traditional intravaginal practices—further sustain its persistence and recurrence [8,9].

From a clinical perspective, the diagnosis and management of vaginal infections in resource-limited settings rely largely on syndromic assessment and basic microscopy rather than molecular diagnostics. In many rural healthcare facilities, nucleic acid amplification tests are unavailable due to financial and infrastructural constraints; consequently, vaginal pH measurement and Gram-stained microscopy with Nugent scoring remain the cornerstone of clinical decision-making. Under these conditions, Nugent classification functions not only as a microbiological grading system but also as a pragmatic clinical tool that integrates microbial imbalance with symptomatology and infection burden. Evaluating how Nugent scores correlate with clinical symptoms, coexisting sexually transmitted infections, and modifiable behavioral factors is therefore of direct relevance to frontline clinicians working in low-resource environments [10].

Despite the growing body of research on vaginal dysbiosis in sub-Saharan Africa, data from Madagascar remain limited, particularly from rural northern regions. Previous investigations conducted by our research group focused primarily on sexually transmitted infection prevalence in this population [11]. The present study expands upon that work by providing a detailed microbiological analysis of vaginal flora composition and Nugent-based classification among women attending a rural second-referral clinic in northern Madagascar.

This study aimed to evaluate the patterns of vaginal bacterial infections among women from northern Madagascar and to determine whether these trends align with those observed in other African countries. Furthermore, it sought to examine how variations in vaginal microflora composition may relate to hygiene behaviors, environmental exposures, and selected socio-demographic factors associated with reproductive health outcomes in resource-limited contexts.

## 2. Materials and Methods

### 2.1. Study Design and Population

This study was conducted in April 2024 during a free medical screening campaign organized at a rural second-referral clinic in Manerinerina, Ambatoboeny District, northern Madagascar. A total of 159 women aged 15–80 years voluntarily participated after providing written informed consent. For participants under 18 years, sampling was performed in the presence of a legal guardian. Sociodemographic, anthropometric, and hygiene-related data were collected through a structured questionnaire administered by trained nurses. Body mass index (BMI) was calculated from anthropometric measurements.

The hygiene section of the questionnaire included three key questions: (1) the direction of genital cleansing during bathing (from anus to vagina or from vagina to anus), (2) the practice of vaginal douching, defined as internal washing or irrigation of the vagina with water or cleansing agents, and (3) the main water source used for bathing and genital hygiene. Water sources were categorized as safe (tap water or protected deep wells), potentially unsafe (unprotected surface wells), and unsafe (river, lake, or stagnant surface water). These variables were later analyzed as potential determinants of vaginal dysbiosis and infection risk.

Clinical examinations and sample collection were performed by trained midwives.

Participants were eligible for inclusion if they had abstained from sexual intercourse for at least seven days, avoided vaginal douching or intravaginal products for at least three days, and were not menstruating or pregnant at the time of sampling. These criteria were introduced to minimize short-term alterations of the vaginal microbiota related to recent sexual activity or intravaginal practices, which could influence vaginal pH measurement and Nugent scoring. All women with confirmed infection or dysbiosis received free treatment and counseling.

### 2.2. Sample Collection and Laboratory Procedures

Vaginal swabs were analyzed immediately in the clinic laboratory. Vaginal pH was measured in situ using standardized narrow-range pH indicator strips (pH 3.8–5.4, Cypress Diagnostics, Hulshout, Belgium) applied to the lateral vaginal wall before sample collection. A wet-mount preparation in 0.9% saline was examined by light microscopy to detect motile *Trichomonas vaginalis*. From the same swab, a smear was prepared on a glass slide, air-dried, heat-fixed, and Gram-stained. Gram-stained smears were evaluated using the Nugent scoring system, which is based on quantification of bacterial morphotypes across multiple oil-immersion fields (1000×): large Gram-positive rods corresponding to *Lactobacillus* spp. (Döderlein bacilli), small Gram-variable rods consistent with *G. vaginalis*/*Bacteroides* spp., and curved Gram-variable rods consistent with *Mobiluncus* spp. Scores for each morphotype (0–4) were summed (range 0–10) and classified as normal flora (0–3), intermediate (4–6), or BV (7–10). The term “cleanliness grades” is used as a clinical shorthand referring to Nugent-based microbiological categories (normal flora, intermediate flora, and bacterial vaginosis) describing the balance of vaginal microbial composition and is used as a synonym for the degree of vaginal microbial balance or dysbiosis. The term does not refer to personal hygiene status. The presence of clue cells (vaginal epithelial cells coated with small Gram-variable coccobacilli) and yeast-like cells compatible with *Candida* spp. was recorded as ancillary microscopic findings but not used in the Nugent score. *Neisseria gonorrhoeae* was assessed on Gram-stained smears by identifying intracellular Gram-negative diplococci within polymorphonuclear leukocytes.

The results for *T. vaginalis* and *N. gonorrhoeae* had previously been partially reported in an earlier publication by the same research team [11]; however, the present analysis focuses on a newly conducted microbiological evaluation of vaginal flora composition and Nugent-based classification, which have not been previously published.

### 2.3. Statistical Analysis

Statistical analyses were performed using Apple Numbers (v13.2, Apple Inc., Cupertino, CA, USA) and Python (v3.12, Python Software Foundation, Wilmington, DE, USA). Descriptive results were expressed as means with standard deviations (SD) or as proportions. Associations between categorical variables were analyzed using the Chi-square test or Fisher’s exact test, where appropriate. A *p*-value of < 0.05 was considered statistically significant.

### 2.4. Declarations/Ethics

The study protocol was reviewed and approved by the Ministry of Public Health of Madagascar (Antananarivo) (No. 108-24/MSANP/SPC; 5 April 2024) and the Ethics Committee of Clinique Médicale Beyzym in Manerinerina, Madagascar. All procedures involving human participants were conducted in accordance with the ethical standards of these committees and the principles outlined in the Declaration of Helsinki (2013 revision). Participation in the study was entirely voluntary. Each participant provided written informed consent prior to enrollment, and for those under 18 years of age, consent was additionally obtained from a parent or legal guardian. Personal identifiers were removed from all data to ensure confidentiality.

## 3. Results

A total of 159 women aged 15–80 years (mean = 36.8 ± 12.8 years) were included in the analysis. The largest age group comprised women aged 25–34 years (43.4%). Regarding nutritional status, 31.4% of participants were underweight (BMI < 18.5), 45.3% had a normal BMI, 15.1% were classified as overweight, and 8.2% had obesity. Most of the women had at least basic education: 17.0% had no formal schooling, 39.6% completed primary education, and 43.4% attended secondary or higher levels (Table 1). Items related to sexual history were included in the questionnaire; however, most participants did not provide answers regarding the number of sexual partners, likely due to the cultural sensitivity of this topic in the studied population.

Based on Nugent’s classification, 68.6% of women presented an abnormal vaginal flora, including 25.2% with intermediate disturbance of vaginal flora (Nugent 4–6) and 43.4% with BV (Nugent 7–10). Only 31.4% of women showed a normal bacterial profile (Nugent 0–3). Normal vaginal pH (≤4.5) was almost exclusively observed in women with Nugent grades 0–3, whereas elevated values predominated in those with intermediate or BV flora (*p* < 0.01) (Table 2).

Sexually transmitted infections (STIs) were also frequent. *T. vaginalis* was detected in 10.7% of women and *N. gonorrhoeae* in 9.4%. Both pathogens were significantly associated with higher Nugent classification (*p* = 0.02 and *p* = 0.04, respectively). *Candida* spp. was identified in 12.0% of samples, representing the third most common pathogen detected in this study. In total, 27.1% of women were affected by at least one infectious pathogen, and 7.5% presented co-infections involving two or more agents.

**Figure 1:**
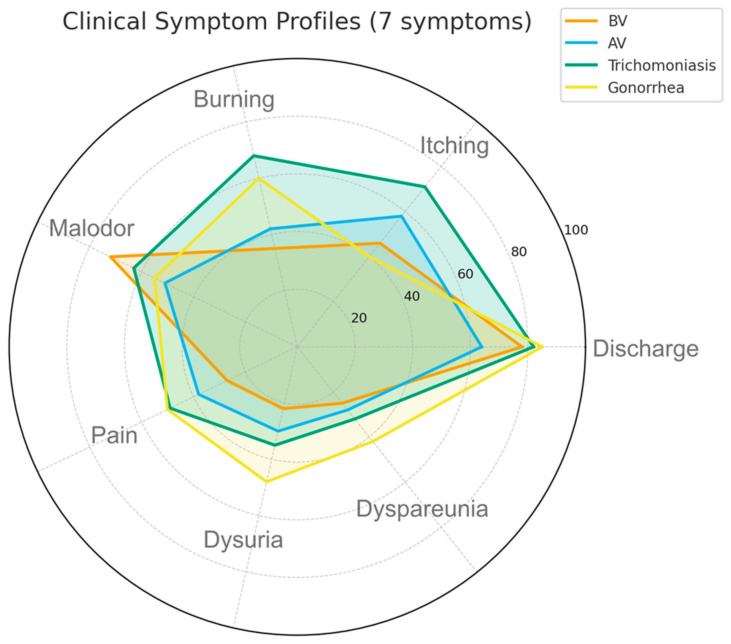
Comparative radar chart showing the prevalence of seven key clinical symptoms among women diagnosed with BV, AV, trichomoniasis, and cervical gonorrhea. Each axis represents one symptom, and the distance from the center reflects its relative frequency within each infection group. Note: BV—bacterial vaginosis; AV—aerobic vaginitis. The chart highlights distinct symptom patterns, with discharge and malodor being most prominent in BV and gonorrhea, whereas itching and burning dominate in trichomoniasis and AV.

Clinical symptoms were reported in 66.7% of women. The most frequent symptoms were vaginal discharge (54.1%), lower abdominal pain (39.6%), and itching (31.4%). The most common symptom pair was vaginal discharge + malodor (r = 0.61), followed by itching + discharge (r = 0.58) and abdominal pain + pelvic pain (r = 0.53), suggesting overlapping presentations of BV and mixed infections. Figure 1 illustrates the comparative distribution of seven key symptoms across infection types, revealing distinct clinical patterns. Discharge and odor were most pronounced in bacterial vaginosis and gonorrhea, whereas itching and burning were predominant in trichomoniasis and aerobic vaginitis. Pain-related symptoms (dysuria and dyspareunia) showed a more heterogeneous distribution, consistent with mixed or secondary inflammatory presentations.

Hygienic practices significantly influenced vaginal flora composition. Women who practiced vaginal douching had a higher rate of dysbiosis (79.1%) compared with those who did not (64.5%; *p* = 0.04). Similarly, poor hygienic practices were associated with increased Nugent scores (74.2% vs. 61.0%; *p* = 0.03). The use of unsafe or probably unsafe water sources also correlated with abnormal flora (*p* = 0.04). Low educational attainment tended to be associated with dysbiosis (71.3% vs. 28.7%), although this trend did not reach statistical significance (*p* = 0.06). Age did not significantly affect the risk of BV (*p* > 0.05), although dysbiosis was slightly more prevalent among younger women (≤30 years) (Table 3).

## 4. Discussion

In this study, the proportion of women with abnormal vaginal flora was high (68.6% overall, including 43.4% with BV according to Nugent’s criteria), dominated by *G. vaginalis* morphotypes and marked by depletion of *Lactobacillus* spp. This depletion was accompanied by significantly elevated vaginal pH, confirming the loss of lactic acid-mediated acidity characteristic of healthy vaginal microbiota (*p* < 0.01). This magnitude aligns with reports from Southern and Eastern Africa, including South Africa, Kenya, Uganda, Tanzania, and Ethiopia, where BV prevalence among women of low socioeconomic status often ranges between 40% and 60% [6,12,13,14,15,16,17,18]. At the same time, it clearly exceeds rates typically documented in high-income countries, where BV affects less than one-third of women of reproductive age [14].

The observed association between higher Nugent scores and the presence of *T. vaginalis* and *N. gonorrhoeae* supports the hypothesis that STI-related inflammation may exacerbate vaginal dysbiosis by disrupting *Lactobacillus*-dominated protection and facilitating anaerobic colonization. This bidirectional interaction suggests that vaginal dysbiosis may not only result from, but also enhance, the transmission of sexually transmitted pathogens [6,9,14]. These findings are consistent with studies from Kenya, Uganda, Tanzania, and South Africa, where co-infection between BV and common STIs is frequent and BV has been linked to increased susceptibility to sexually transmitted pathogens [12,13,14,15]. Conversely, *Candida* spp. (detected in 12.0% of samples) likely reflects local dysbiosis and altered vaginal ecology rather than sexual transmission per se [14,16,17,18].

The use of Gram-stained smears and Nugent scoring remains the most practical and standardized diagnostic tool for field conditions, particularly in low-resource settings where molecular testing is unavailable, as confirmed by studies from Ethiopia, Ghana, and the Democratic Republic of the Congo [6,16,17,18]. Previous Malagasy investigations—both classical and contemporary—have similarly highlighted the heavy burden of lower reproductive tract infections and the diagnostic importance of simple microscopy-based algorithms in primary healthcare [19,20,21]. Taken together, these observations place northern Madagascar within the broader sub-Saharan epidemiological landscape—marked by a high prevalence of BV and dysbiosis, frequent overlapping symptoms, and strong interconnections between BV and sexually transmitted infections [6,12,13,14,15,16,17,18,21].

Clinical manifestations observed in this cohort were broadly consistent with findings from other sub-Saharan African populations. In our study, discharge and malodor predominated in BV and gonorrhea, while itching and burning were more characteristic of trichomoniasis and aerobic vaginitis. Similar symptom distributions have been reported in cohorts from South Africa and Kenya, where discharge and odor were the most frequent clinical complaints among women with BV and mixed infections [12,13]. Studies from Tanzania and Ethiopia also indicated that itching and burning were more indicative of inflammatory or protozoal etiologies rather than bacterial vaginosis [15,16]. These findings suggest that, although vaginal discharge remains a non-specific marker, the combination of odor and discharge may serve as a useful clinical indicator of BV in settings lacking molecular diagnostics, while itching and burning point toward trichomoniasis or AV. This interpretation aligns with broader evidence that symptom-based differentiation, despite its limitations, can guide empirical management where laboratory resources are scarce [6,9].

Vaginal douching emerged as a significant behavioral determinant of vaginal dysbiosis in our cohort, with 79.1% of women who practiced douching presenting with abnormal flora compared to 64.5% among non-douchers (*p* = 0.04). This practice remains widespread across various low- and middle-income settings, often motivated by perceived hygiene benefits, odor control, or infection prevention, despite limited awareness of its adverse effects [20,22,23]. Beyond epidemiological associations, the biological mechanisms underlying this relationship have been increasingly elucidated. Evidence from previous studies indicates that intravaginal cleansing, particularly when using acidic or plant-based substances, may disrupt the vaginal microbiota and increase susceptibility to sexually transmitted infections. In Haiti, such practices were associated with a higher prevalence of high-risk human papillomavirus (HPV) infection [24], while among female sex workers in China, vaginal douching correlated with increased risks of HIV and HSV-2 acquisition [25]. Cultural norms and social influences, including advice from healthcare personnel, appear to sustain these behaviors, underscoring the importance of culturally tailored education and preventive strategies [20,22].

From a pathophysiological perspective, vaginal douching alters vaginal pH, disrupts *Lactobacillus* dominance, and compromises mucosal integrity, thereby facilitating BV and other opportunistic infections [26,27]. Data from intervention studies suggest that even when counseling reduces the frequency of intravaginal cleansing, the prevalence of BV remains persistently high [27]. The magnitude of risk likely depends on the type and intensity of the practice, with non-water or irritant substances being particularly harmful [27]. Collectively, available evidence supports that vaginal douching is a culturally embedded yet detrimental hygiene behavior that adversely affects vaginal ecology and contributes to the burden of vaginal infections, especially in resource-limited environments with restricted access to accurate reproductive health education [20,22,23,24,25,26,27].

Taken together, these findings illustrate the multifactorial nature of vaginal dysbiosis in this rural Malagasy population, where microbial, behavioral, and environmental determinants intersect to shape women’s reproductive health. The interplay between abnormal vaginal flora, sexually transmitted infections, and hygiene practices reflects a complex ecological equilibrium that remains insufficiently understood in low-resource contexts. The near-significant association with lower education level may indicate unmeasured contextual factors but requires confirmation in larger cohorts. Interpretation of behavioral pathways remains limited due to incomplete sexual history data, including the number of sexual partners. Notably, our previous population-based analysis of morbidity patterns in the Ambatoboeny District demonstrated that women face statistically lower access to healthcare services compared with men, a disparity that adds another layer of vulnerability in the context of reproductive tract infections [19]. This finding suggests that frequent vaginal infections may disproportionately affect individuals facing barriers to diagnosis and treatment.

### 4.1. Implications for Practice in Resource-Limited Settings

The findings of this study suggest that general education level alone does not necessarily translate into better prevention of vaginal dysbiosis or improved genital hygiene practices. In rural areas such as northern Madagascar, health promotion efforts should therefore move beyond formal education and focus on targeted, culturally adapted interventions. Primary- and secondary-level health facilities could play a pivotal role in this process by integrating educational sessions into routine clinical encounters. Such programs should emphasize practical aspects of vaginal hygiene, awareness of risk behaviors such as douching, and early recognition of infection symptoms. Strengthening the educational function of health centers could represent a cost-effective, sustainable approach to reducing the burden of vaginal infections in resource-constrained communities.

Beyond education, improving access to safe water and basic hygiene supplies should be considered a core component of reproductive health interventions. Our findings show that women relying on unsafe water sources faced a significantly higher prevalence of vaginal dysbiosis. Ensuring the availability of clean water for personal hygiene and promoting the use of mild, non-irritant soaps could help prevent recurrent infections and reduce dependence on empiric antibiotic treatment. These measures, while simple, address one of the structural determinants of women’s health that extends beyond the clinic setting.

Finally, the use of low-cost microscopy for Nugent scoring demonstrates a feasible diagnostic approach for rural clinics lacking molecular infrastructure. The material cost of a microscopy-based evaluation is approximately 0.5 EUR per sample, which remains affordable even in low-income settings such as Madagascar, where the monthly minimum wage is around 274,700 MGA (≈ 55 EUR). Incorporating such screening into reproductive health services could improve early detection, enable treatment monitoring, and provide epidemiological data for local health authorities. This integration of low-cost diagnostics, community education, and improved hygiene infrastructure represents a pragmatic and scalable model for strengthening women’s reproductive health in low-resource environments.

A practical training framework derived from these findings is provided in Appendix A, illustrating how key components of reproductive health education can be implemented in resource-limited clinical settings through community-based training and hygiene promotion sessions. For broader accessibility, the material is presented in both English and Malagasy.

### 4.2. Limitations

This study has several limitations that should be acknowledged. First, the relatively small sample size reflects the inherent challenges of recruiting women for reproductive health research in rural Madagascar. Discussions about sexuality and genital symptoms remain culturally sensitive and often stigmatized, leading many women to avoid clinical consultations or decline participation. In many Malagasy communities, reproductive health is still approached within a paternalistic framework in which men’s and children’s health needs are prioritized over women’s [28,29,30,31]. Recruitment during a free screening campaign may have introduced selection bias toward symptomatic or health-seeking women; therefore, prevalence estimates should be interpreted cautiously, as they may not fully represent the general community and could potentially overestimate symptomatic conditions.

The diagnosis of *T. vaginalis* and *N. gonorrhoeae* was based exclusively on light microscopy of vaginal smears, as molecular diagnostics are unavailable across the region. Although microscopy has limited sensitivity—particularly for *N. gonorrhoeae*—the presence of intracellular Gram-negative diplococci in symptomatic women is considered a reliable indicator of infection according to the diagnostic algorithms endorsed by the Malagasy Ministry of Public Health [21,32]. Reported sensitivities for wet-mount microscopy in detecting *T. vaginalis* range between 40% and 70%, with specificity up to 100% [33,34]. Nevertheless, microscopy-based identification may introduce misclassification bias compared with nucleic acid amplification tests.

No multivariable modelling was performed due to the modest sample size and the exploratory nature of the study; therefore, potential confounding between factors such as education level, hygiene practices, and water source cannot be excluded. Future studies with larger cohorts should incorporate multivariate analyses to better assess independent associations.

Finally, although broader structural and social determinants of health are discussed in the context of regional literature, these factors were not directly measured within the present study and should therefore be interpreted as contextual considerations rather than causal inferences.

## 5. Conclusions

This study demonstrates that vaginal dysbiosis and bacterial vaginosis are highly prevalent among women in northern Madagascar, affecting nearly seven in ten participants. The predominance of *Gardnerella*-like morphotypes and depletion of *Lactobacillus* spp. highlight a pattern consistent with other low-income regions of sub-Saharan Africa, yet the data also underscore the scarcity of microbiological surveillance in rural Madagascar, where reproductive tract infections remain underdiagnosed and undertreated.

Despite relying solely on microscopy-based diagnostics, this study provides valuable epidemiological evidence from a population rarely represented in international literature. The findings underline the practical utility of Nugent scoring as a low-cost, field-appropriate tool for assessing vaginal health in environments lacking molecular infrastructure. Beyond their diagnostic role, these microscopy-based evaluations can serve as indirect indicators of community-level determinants—education, hygiene behavior, and access to clean water—that collectively shape women’s reproductive outcomes.

Future research should integrate molecular methods and longitudinal follow-up to better characterize microbial resilience, treatment response, and recurrence of dysbiosis.

Ultimately, Nugent-based microbiological classifications observed in this study may serve as indirect indicators of broader reproductive health determinants shaped by environmental and socio-demographic conditions in resource-limited settings.

## Figures and Tables

**Table 1:** General characteristics of the study population (*n* = 159).

Variable	*n* (%)
Age, mean (±SD), years	36.8 ± 12.8
Age group 25–34 y	69 (43.4)
BMI underweight (<18.5)	50 (31.4)
BMI normal (18.5–24.9)	72 (45.3)
BMI overweight (25.0–29.9)	24 (15.1)
BMI obese (≥30)	13 (8.2)
No formal education	27 (17.0)
Primary education	63 (39.6)
Secondary + higher	69 (43.4)

**Table 2:** Nugent’s classification distribution and infection prevalence.

Category	*n* (%)	Description
Normal flora (Nugent 0–3)	50 (31.4)	Balanced microbiota
Intermediate flora (Nugent 4–6)	40 (25.2)	Partial dysbiosis
BV (Nugent 7–10)	69 (43.4)	Established BV
Any dysbiosis (4–10)	109 (68.6)	–
*T. vaginalis*	17 (10.7)	STI
*N. gonorrhoeae*	15 (9.4)	STI
*Candida* spp.	19 (12.0)	Non-STI ^1^ fungal infection
≥1 pathogen detected	43 (27.1)	–

^1^ STIs: sexually transmitted infections; BV: bacterial vaginosis.

**Table 3:** Factors associated with abnormal vaginal flora (univariate analysis).

Variable	Dysbiosis %	Normal %	*p*-Value	Test Used
Vaginal pH > 4.5	88.7	11.3	0.01	Chi-square test
Poor hygiene practices	74.2	25.8	0.03	Chi-square test
Vaginal douching (yes)	79.1	20.9	0.04	Chi-square test
Unsafe water source	77.8	22.2	0.04	Chi-square test
Low education (none/primary)	71.3	28.7	0.06	Chi-square test
Age ≤ 30 y	70.5	29.5	ns	Chi-square test
Trichomoniasis	85.5	15.0	0.02	Fisher’s exact test
Cervical gonorrhea	82.0	18.0	0.04	Fisher’s exact test

*p*-values < 0.05 were considered statistically significant.

**Figure A1:**
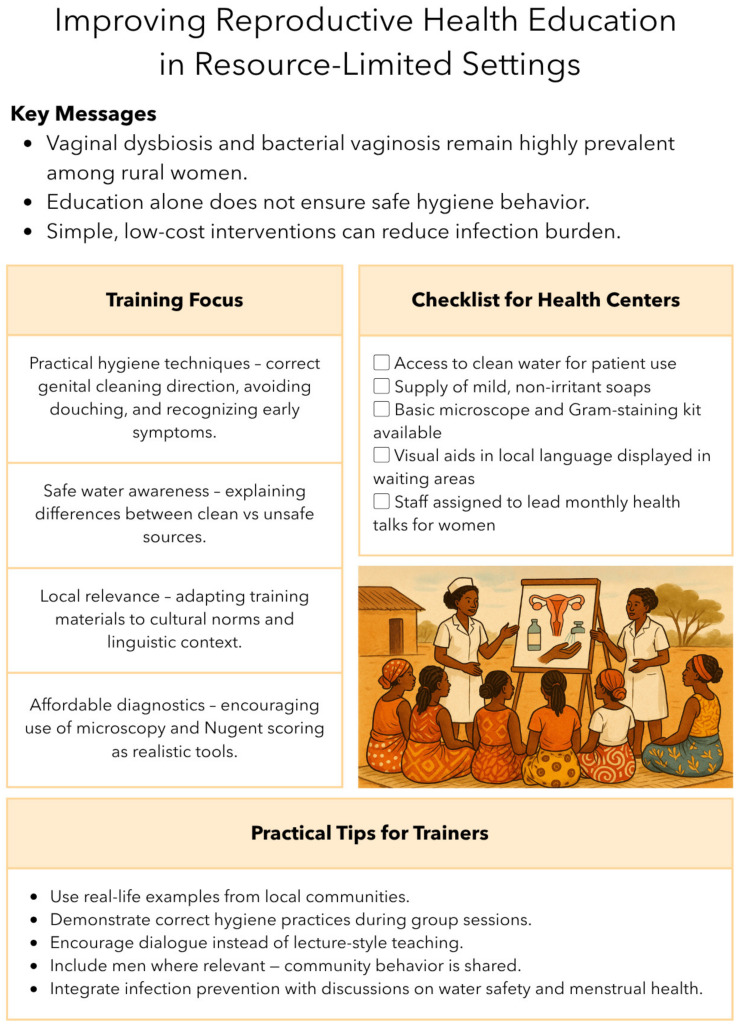
Improving reproductive health education in resource-limited setting—a practical training framework [English Version].

**Figure A2:**
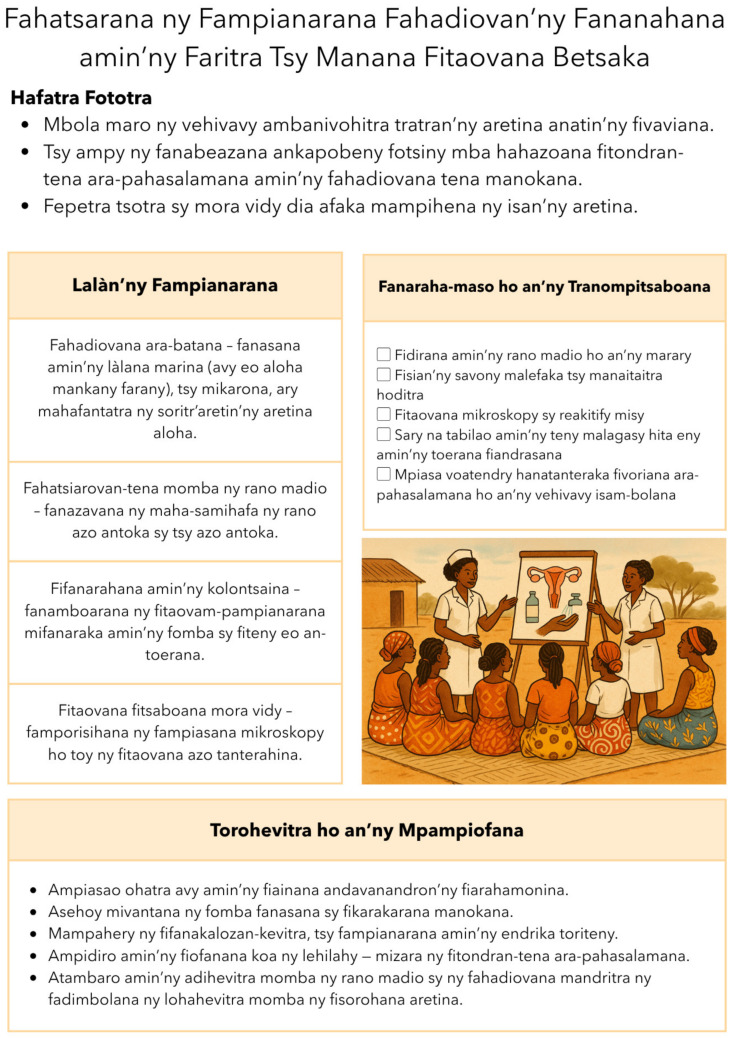
Improving reproductive health education in resource-limited setting—a practical training framework [Malagasy Version].

## Data Availability

The data presented in this study are available upon request from the corresponding author.
